# Special Issue “Antiviral Drugs Discovery”

**DOI:** 10.3390/ijms27188055

**Published:** 2026-09-10

**Authors:** Sining Tao, Zhenzhen Zhou, Xinyong Liu, Dongwei Kang

**Affiliations:** Department of Medicinal Chemistry, Shandong Key Laboratory of Druggability Optimization and Evaluation for Lead Compounds, Key Laboratory of Chemical Biology (Ministry of Education), School of Pharmaceutical Sciences, Shandong University, 44 West Culture Road, Jinan 250012, China; 202436849@mail.sdu.edu.cn (S.T.); zhouzhensdu@163.com (Z.Z.)

Emerging and re-emerging viral pathogens pose a persistent and significant threat to global public health and socioeconomic stability [1,2]. A wide spectrum of viruses, including respiratory syncytial virus (RSV) infection, severe acute respiratory syndrome coronavirus 2 (SARS-CoV-2), and Zika virus (ZIKV), have triggered large-scale epidemics repeatedly worldwide, resulting in massive numbers of infections and deaths, along with substantial social and economic losses [3,4,5,6,7]. Currently, antiviral drugs available for clinical use generally suffer from narrow antiviral spectra and limited therapeutic efficacy [8,9]. Particularly concerning is the rapid and widespread emergence of drug-resistant viral strains, which severely restrict the clinical utility of existing treatments and leave people worldwide vulnerable to repeated viral outbreaks [10]. Under these circumstances, developing new antiviral drugs with entirely novel targets and mechanisms—characterized by high efficiency and broad-spectrum activity—has become a top research priority for modern pharmacology [1,3].

This Special Issue systematically elaborates on the current challenges in antiviral drug research and innovative research strategies. We focus on highly pathogenic viruses that pose a serious threat to global public health, including RSV, SARS-CoV-2, and ZIKV. In addition, we focus on innovative antiviral research strategies, including the exploration of novel drug targets, computer-aided drug design (CADD), host immune modulation pathways, and dual-target drug candidates derived from natural products. These approaches help overcome key drawbacks of conventional drug discovery approaches, such as low screening throughput and the risk of drug resistance caused by mutations. This Special Issue provides new insights into developing next-generation antiviral drugs that are highly effective and feature broad-spectrum activity, offering solutions to future viral outbreaks and emerging, unknown viral infections.

Natural products are valuable resources for discovering antiviral candidates. Many molecules derived from natural sources can simultaneously target viral targets and host inflammatory pathways, making them excellent candidates for the development of antiviral–anti-inflammatory dual-target drugs [11,12,13]. RSV belongs to the *paramyxoviridae* family and is an important pathogen that causes lower respiratory tract infections in infants, elderly individuals, and immunocompromised populations [14,15]. Currently, only ribavirin is clinically approved for the treatment of respiratory syncytial virus. However, due to its severe toxic side effects, ribavirin is prohibited for use in children under the age of three [16].

A research report in this issue presents a dual-target natural-product-based drug candidate that simultaneously targets RSV F protein and arachidonate 15-lipoxygenase (15-LOX) [17] used the Traditional Chinese Medicine Systems Pharmacology Database (TCMSP) to screen natural products. Using druglikeness evaluation, molecular docking, ADME assessment, and molecular dynamics simulation, they screened 13,131 natural compounds and obtained 31 potential dual-target candidate compounds with favorable druglikeness. Among them, rhoeadine was identified, a compound that maintains a stable binding conformation with both targets during the simulation process. Rhoeadine exhibits significant activity against RSV (EC_50_ = 1.82 μM) and low cytotoxicity (CC_50_ = 34.50 μM). This compound can act at the early stage of RSV infection, not only by directly inhibiting viral replication but also by exerting anti-inflammatory effects via regulating host 15-LOX.

SARS-CoV-2, a member of the family *Coronaviridae*, is a highly pathogenic virus characterized by robust human-to-human transmissibility and continuous mutational capacity [18]. It triggered the global COVID-19 pandemic in 2019, which resulted in hundreds of millions of infections and millions of deaths and inflicted a severe blow to global public health security [19]. The virus readily generates drug-resistant variants via gene mutations, which attenuates the therapeutic efficacy of conventional antiviral agents [20,21]. Most mainstream drugs inhibit SARS-CoV-2 by targeting the virus’s conserved functional enzymes, such as the main protease (M^pro^) and RNA-dependent RNA polymerase (RdRp) [22]. However, the singularity of these targets gives rise to prominent drug resistance risks and renders these drugs vulnerable to ongoing viral mutations [23]. Accordingly, exploring novel targets and expanding the target spectrum lead to critical breakthroughs for developing next-generation inhibitors against coronavirus.

A study in this Special Issue focuses on the helicase protein non-structural protein 13 (nsp13), a core functional protein of the replication–transcription complex [24] used an ultra-high-throughput nucleic acid unwinding assay to screen multiple drugs and bioactive compounds approved by the U.S. Food and Drug Administration (FDA) and identify a series of nsp13 inhibitors. Distinct from conventional nsp13 inhibitor, these compounds present a novel allosteric mode of action, suppressing helicase function without perturbing substrate binding or ATP hydrolysis. The IC_50_ values of this series of compounds range from 1.4 to 10 μM, and they exhibit antiviral activity against infectious SARS-CoV-2 in various cell lines, providing an important molecular basis for subsequent structure optimization.

Since the outbreak of the COVID-19 pandemic, SARS-CoV-2 has undergone frequent genetic mutations and continuously generated multiple variant strains, including Alpha, Delta, and Omicron [19,25]. These variants have greatly enhanced viral transmissibility and immune evasion capacity and notably increased drug resistance against current therapeutics, imposing great pressure on clinical treatment [26]. Conventional drug development is characterized by long cycles, high costs, and low efficiency in discovering active molecules and cannot respond to viral variation and drug resistance crises in a rapid manner. Structure-based computational virtual screening features high efficiency, low cost, and high-throughput capacity [27,28], enables precise identification of high-affinity small-molecule candidates based on target protein structures, and substantially shortens the development period of new drugs.

To develop novel SARS-CoV-2 inhibitors against evolving drug-resistant variants, Liu et al. designed a fragment-based multilevel virtual screening strategy based on the reported lead compound WU-04 [29]. Fifteen hit compounds were obtained after screening and chemical synthesis. Among them, compound A9 exhibited the most potent antiviral activity with an EC_50_ of 0.8 μM, comparable to the marketed drug nirmatrelvir (EC_50_ = 0.123 μM), making it a highly promising anti-SARS-CoV-2 lead compound. This hierarchical computational screening strategy enriches bioactive lead compounds and provides a powerful technical solution for antiviral drug discovery.

*Flavivirus* comprises a family of vector-borne, highly pathogenic RNA viruses, including ZIKV, dengue virus (DENV), and Japanese encephalitis virus (JEV) [30]. Among them, ZIKV is a highly contagious disease that poses a serious threat to public health in tropical and subtropical regions worldwide [31]. However, no specific antiviral drugs for this disease or completely effective vaccines are currently available for clinical intervention [32,33]. Most candidates possess only single-target profiles with poor broad-spectrum activity [34,35]. Therefore, developing safe and potent novel ZIKV inhibitors bears considerable clinical significance.

Murineddu and colleagues [36] synthesized 23 pyrrole-based tricyclic derivatives bearing bulky amine moieties and evaluated their antiviral activities. Among them, compound 2 g exhibited excellent activity against ZIKV and extremely low cytotoxicity (EC_50_ and CC_50_ values of 0.4 μM and 230.5 μM, respectively). Mechanism studies have revealed that compound 2 g primarily downregulated the levels of viral non-structural protein 5 (NS5) protein, thus impairing viral protein function. Additionally, this series of compounds showed an inhibitory effect on SARS-CoV-2, and this study provides a high-quality lead scaffold for the structural optimization of novel ZIKV inhibitors.

Most antiviral drug discovery efforts focus on viral structural or functional proteins. Such agents rely heavily on conserved viral structures and tend to develop drug resistance via viral gene mutations, making them poorly suited to address cross-virus infections and ongoing viral evolution [3]. In contrast, host-directed immunomodulatory strategies target highly conserved human host pathways [37], which are less likely to elicit drug resistance, can simultaneously suppress viral replication, modulate immune dyshomeostasis, alleviate inflammatory injury, and offer broad-spectrum antiviral advantages [38]. These strategies have become a research hotspot for the treatment of emerging and re-emerging viral infections.

Schiffmann et al. [39] systematically investigated the immunomodulatory properties of two eukaryotic translation initiation factor eIF4A inhibitors: pateamine A (PatA) and its derivative des-methyl des-amino pateamine A (DMDA). Their study revealed that PatA potently inhibits B-cell activation, whereas DMDA exhibits improved safety but suffers from insufficient cell membrane permeability. Their work suggests that the development of host-targeted antiviral immunotherapeutics requires balancing therapeutic potential against druggability limitations.

Overall, the research articles on antiviral drug discovery featured in this Special Issue mainly focus on the discovery of antiviral drugs as well as on studying interactions between antiviral drugs and the immune system. These notable contributions demonstrate the development trends of contemporary antiviral drug research: firstly, the increasing range of targets and antiviral strategies, as research is increasingly conducted on novel targets such as helicases and host immune regulation; secondly, the sources of lead compounds are becoming increasingly diverse, including the derivatives of natural products and artificially synthesized small-molecule scaffolds; thirdly, computer-aided drug design techniques such as molecular docking and multilevel virtual screening playing important roles in drug-screening systems.

Looking ahead, numerous challenges remain in antiviral drug development. Although candidate molecules have shown promising results in cell experiments, their pharmaceutical properties still require further in vivo validation and optimization. Compounds derived from natural products should undergo further structural modifications and safety assessments, and host-directed immunomodulation strategies still need to address issues such as poor cell permeability and side effects. Furthermore, we should enhance research on broad-spectrum antiviral drugs to better respond to emerging viral outbreaks.

Hereby, we sincerely thank all authors for their admirable work, and we extend our gratitude to all review experts for their careful evaluations, which have effectively enhanced the academic quality of the papers in this Special Issue, as well as to the journal editors and staff for their efficient support. We hope that the research findings presented here serve as references for future studies, thereby promoting innovative development in this field.
